# Supplementary material for: Utility and cost-effectiveness of LiverMultiScan for MASLD diagnosis: a real-world multi-national randomised clinical trial
Source: Commun Med (Lond). 2025 Mar 18;5:74. doi: 10.1038/s43856-025-00796-9 (PMC11920111; doi:10.1038/s43856-025-00796-9)

Multiparametric MRI increases rate of MASLD diagnosis with fewer specialist appointments and interventions: a real-world multi-national randomised clinical trial

Elizabeth Shumbayawonda, Marika French, Jane Elizabeth Carolan, Cayden Beyer, Paula Lorgelly, Dimitar Tonev, Rajarshi Banerjee, Michael H Miller, Christopher D Byrne, Janisha Patel, Saima Ajaz, Kosh Agarwal, Johanna Backhus, Minneke J Coenraad, Jelte J Schaapman, Andrew Fraser, Miguel Castelo Branco, Stephen Barclay, Matthias M. Dollinger, Daniel J Cuthbertson, Daniel Forton, Hildo J Lamb

Supplementary tables and figures

Supplementary table 1: inclusion and exclusion criteria
Supplementary table 2: CPI Hospital Index for Germany, The Netherlands and Portugal (OECD)
Supplementary table 3: MRI parameters for acquiring mpMRI

Supplementary Fig 1: Aggregate short-term costs incurred in the management of patients in the imaging (SoC + mpMRI) and the SoC arm (A) without and (B) with MRI costs. MRI costs incurred reflect the combined MRI scan tariff and the mpMRI cost.
Supplementary Fig 2: Regional aggregate number of visits with health care practitioners (general practitioner, specialist outside hospital visit, specialist at hospital visit and therapist) and patient assessments (blood tests, biopsy and ultrasound testing) between the imaging arm (SoC + mpMRI) and standard of care arms.

Supplementary table 1: inclusion and exclusion criteria

Inclusion criteria	Exclusion criteria
Patients had to either have either:<br>(1)<br>• Elevated liver enzymes (ALT, AST or GGT ≥1.5 x upper limit of normal and ALT,<br>• AST ≤5 x upper limit of normal) up to 1 year prior to patient recruitment<br>• OR imaging suggestive of Fatty liver disease up to 3 years prior to patient recruitment,<br><br>Or<br><br>(2)<br>• Have the presence of ≥3 of the following criteria: insulin resistance or type 2 diabetes mellitus,<br>• Obesity (BMI >30 or waist-to-hip ratio >1.00 for men / >0.85 for women),<br>• Hypertension (≥130/85 mmHg),<br>• Elevated triglycerides (≥1.7 mmol/l), or<br>• Low HDL-cholesterol (<1.05 mmol/l for men / <1.25 mmol/l for women).	• Contraindication to MRI,<br>• Proven liver disease other than NAFLD,<br>• Liver transplantation, clinical signs of chronic liver failure (variceal bleeding,<br>• Ascites, overt encephalopathy), pregnancy,<br>• Alcohol over-use/abuse as determined by local guidelines,<br>• Known malignant liver tumours and those with any malignancy with life expectancy <36 months,<br>• Heart failure NYHA stages II-IV,<br>• Severe mental illness.<br>• Any other cause of disease, including a significant disease or disorder which, in the opinion of the investigator, may either put the participant at risk because of participation in the study, or may influence the result of the study, or the participant’s ability to participate in the study

Supplementary table 2: CPI Hospital Index for Germany, The Netherlands and Portugal (OECD)

Country	2017	2018	2019	2020
Consumer Prices Hospital index				
Germany	1.4	0.9	1.0	
The Netherlands	0.6	1.4	2.5	
Consumer Prices Hospital index: Organisation for Economic Co-operation and Development				
Portugal	0.4	1.1	0.8	
United Kingdom	2.5	2.5	2.6	
Office of National Statistics, CPI Health Value for United Kingdom				
United Kingdom			109.1	112.1

Supplementary table 3: MRI parameters for acquiring mpMRI

Field of view (mm3)	440 x 330 x 100
Reconstruction voxel size (mm)	1.15 x 1.15
Slice thickness (mm)	8
Slice gap (mm)	7
Slices	5
Parallel imaging factor	2
Repetition time (ms)	2.42
Echo time (ms)	1.05
Flip angle (°)	35
Acquisition duration	60 seconds
Respiratory compensation	5 breath-holds

Supplementary Fig 1: Aggregate short-term costs incurred in the management of patients in the imaging (SoC + mpMRI) and the SoC arm (A) without and (B) with MRI costs. MRI costs incurred reflect the combined MRI scan tariff and the mpMRI cost.

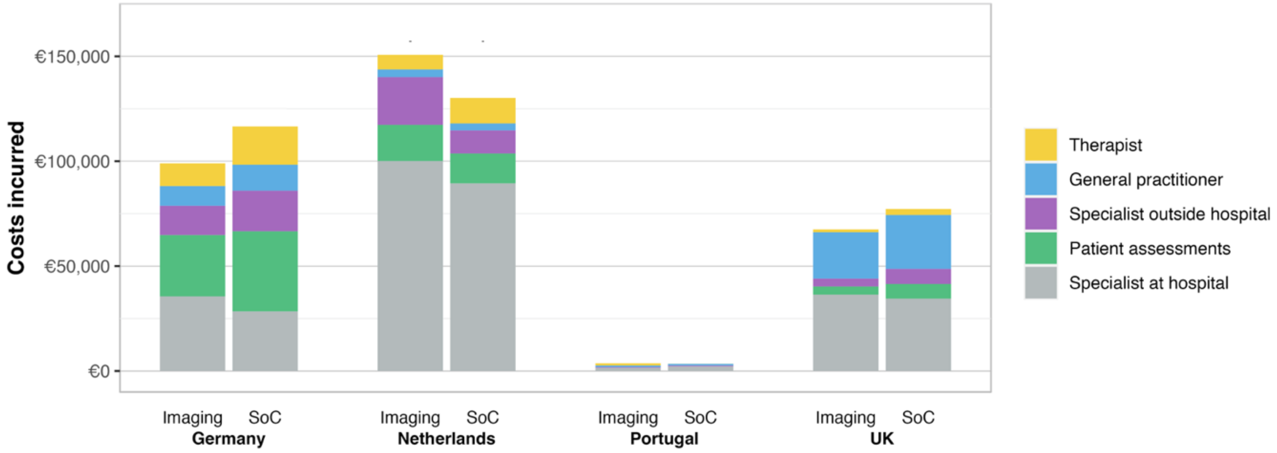

A

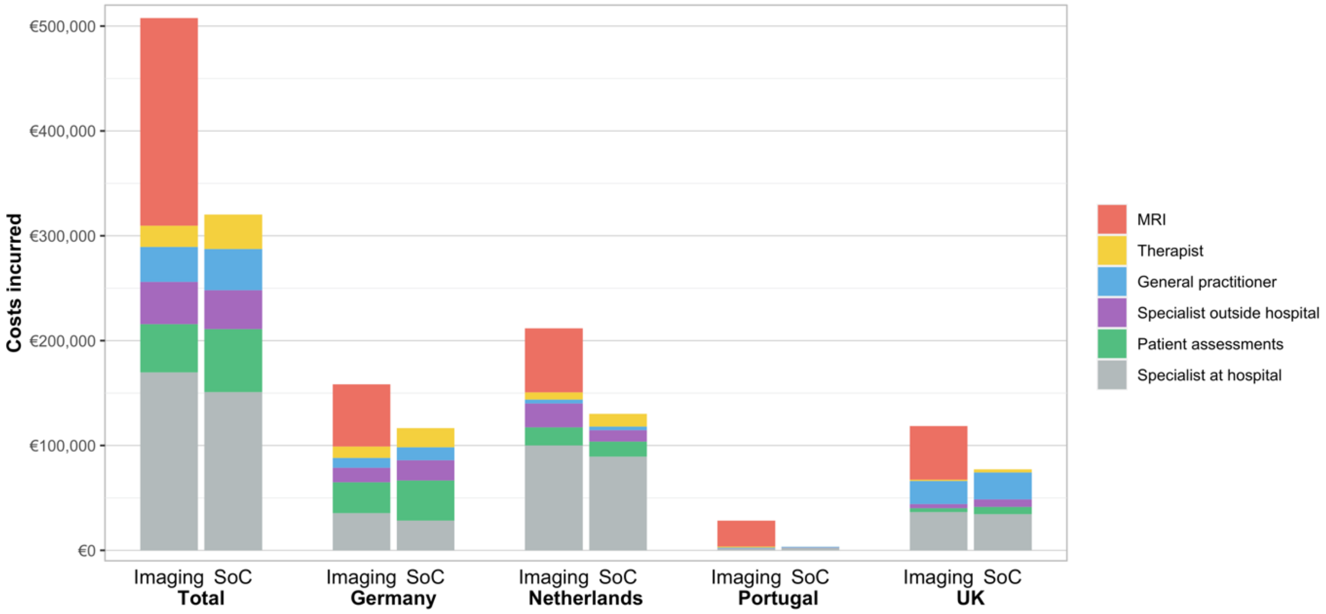

B

Supplementary Fig 2: Regional aggregate number of visits with health care practitioners (general practitioner, specialist outside hospital visit, specialist at hospital visit and therapist) and patient assessments (blood tests, biopsy and ultrasound testing) between the imaging arm (SoC + mpMRI) and standard of care arms.

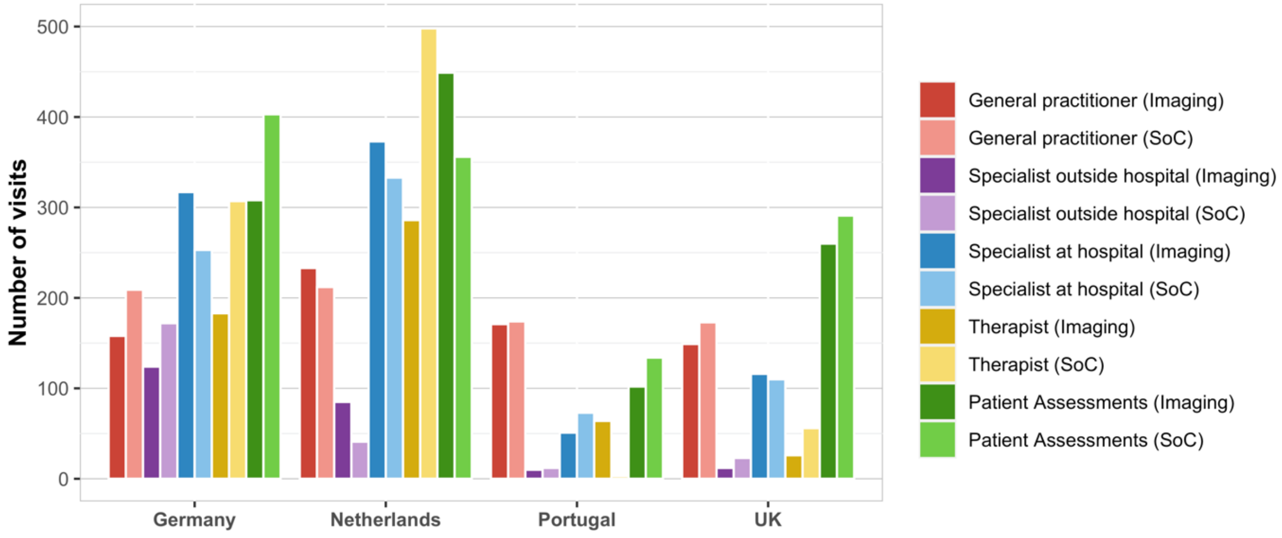

Supplement: Supplementary file 2 — Supplementary information [file 43856_2025_796_MOESM2_ESM.pdf]
