# Supplementary material for: Utility and cost-effectiveness of LiverMultiScan for MASLD diagnosis: a real-world multi-national randomised clinical trial
Source: Commun Med (Lond). 2025 Mar 18;5:74. doi: 10.1038/s43856-025-00796-9 (PMC11920111; doi:10.1038/s43856-025-00796-9)
Supplement: Supplementary file 3 — REPORTING SUMMARY [file 43856_2025_796_MOESM3_ESM.pdf]

nature portfolio

Corresponding author(s): Elizabeth Shumbayawonda

Last updated by author(s): Feb 21, 2025

Reporting Summary

Nature Portfolio wishes to improve the reproducibility of the work that we publish. This form provides structure for consistency and transparency in reporting. For further information on Nature Portfolio policies, see our Editorial Policies and the Editorial Policy Checklist.

Statistics

For all statistical analyses, confirm that the following items are present in the figure legend, table legend, main text, or Methods section.

n/a	Confirmed	
☐	☒	The exact sample size (*n*) for each experimental group/condition, given as a discrete number and unit of measurement
☐	☒	A statement on whether measurements were taken from distinct samples or whether the same sample was measured repeatedly
☐	☒	The statistical test(s) used AND whether they are one- or two-sided<br>*Only common tests should be described solely by name; describe more complex techniques in the Methods section.*
☒	☐	A description of all covariates tested
☐	☒	A description of any assumptions or corrections, such as tests of normality and adjustment for multiple comparisons
☐	☒	A full description of the statistical parameters including central tendency (e.g. means) or other basic estimates (e.g. regression coefficient) AND variation (e.g. standard deviation) or associated estimates of uncertainty (e.g. confidence intervals)
☒	☐	For null hypothesis testing, the test statistic (e.g. *F*, *t*, *r*) with confidence intervals, effect sizes, degrees of freedom and *P* value noted<br>*Give P values as exact values whenever suitable.*
☒	☐	For Bayesian analysis, information on the choice of priors and Markov chain Monte Carlo settings
☒	☐	For hierarchical and complex designs, identification of the appropriate level for tests and full reporting of outcomes
☐	☒	Estimates of effect sizes (e.g. Cohen's *d*, Pearson's *r*), indicating how they were calculated

Our web collection on statistics for biologists contains articles on many of the points above.

Software and code

Policy information about availability of computer code

Data collection	All mpMR images were obtained using a non-contrast abdominal MRI scan following the LiverMultiScan image acquisition protocol. LiverMultiScan is a proprietary commercial multiparametric MRI tool produced by Perspectum Ltd (Oxford, UK)) with 510k clearance, UKCA, and CE marking.
Data analysis	Statistical analyses were performed using R version 1.4.1103. All code used for statistical analyses were generated to analyse the data collected in this study.

For manuscripts utilizing custom algorithms or software that are central to the research but not yet described in published literature, software must be made available to editors and reviewers. We strongly encourage code deposition in a community repository (e.g. GitHub). See the Nature Portfolio guidelines for submitting code & software for further information.

Data

Policy information about availability of data

All manuscripts must include a data availability statement. This statement should provide the following information, where applicable:

- Accession codes, unique identifiers, or web links for publicly available datasets
- A description of any restrictions on data availability
- For clinical datasets or third party data, please ensure that the statement adheres to our policy

The data and analytic methods used in this study remain the property of the study sponsors. All deidentified participant data may be made available to other researchers upon request following permission, investigator support and following a signed data access agreement.

Human research participants

Policy information about studies involving human research participants and Sex and Gender in Research.

Reporting on sex and gender	Findings reported herein apply to both sexes. Sex was considered in the study design, and it was based on biological attribute (i.e. male or female). In this study 56% of those recruited were male (225 in the imaging arm, 228 in the standard of care arm; table 1). Sex was determined on self-reporting and confirmation on clinical notes, gender (shaped by social and cultural circumstances) was not reported. No sex specific analyses were performed as there are no reported clinical differences in the disease presentation between sexes.
Population characteristics	All recruited participants were adults aged 18-75 years and were due to undergo evaluation for suspicion of MASLD/MASH Participants had an average age of 53 years (±13). 42% of participants (43.7% in the imaging arm, 41.1% in the standard of care arm) had type 2 diabetes at recruitment (table 1).
Recruitment	For inclusion patients had to have either elevated liver enzymes, imaging suggestive of fatty liver disease, or have the presence of 3 or more of the following criteria: insulin resistance or type 2 diabetes mellitus, obesity, hypertension, elevated triglycerides, or low HDL-cholesterol. Exclusion criteria included contraindication to MRI, proven liver disease other than NAFLD, liver transplantation, clinical signs of chronic liver failure, pregnancy, alcohol over-use/abuse and any other cause of disease, which in the opinion of the investigator, may affect the participant's ability to participate in the study.
Ethics oversight	The protocol, informed consent form, participant information sheet, and any proposed advertising material was submitted to each host institution's appropriate research ethics committee for written approval; a favorable (and granted) response was received in Ulm (198/17), Leiden (P17.076), Coimbra (CE-030/2017), and UK (18/SC/0725).

Note that full information on the approval of the study protocol must also be provided in the manuscript.

Field-specific reporting

Please select the one below that is the best fit for your research. If you are not sure, read the appropriate sections before making your selection.

☐ Life sciences ☒ Behavioural & social sciences ☐ Ecological, evolutionary & environmental sciences

For a reference copy of the document with all sections, see nature.com/documents/nr-reporting-summary-flat.pdf

Behavioural & social sciences study design

All studies must disclose on these points even when the disclosure is negative.

Study description	This was a prospective, observational, randomised control trial. Cross sectional quantitative data were collected
Research sample	The study sample comprised of adults who were due to undergo evaluation for suspicion of MASLD. Recruited participants had a mean age of 53 years, 42% had type 2 diabetes
Sampling strategy	In a study by Blake et al (2016), the use of LiverMultiScan was found to result in an 18% decrease in the use of biopsy. Adopting a conservative target of identifying a 14% decrease across different regions, each randomization arm is required to have 402 patients to maintain statistical significance with more than 80% power and show a difference in proportion of patients having consultations between the 2 arms. Due to the size of the trial, assuming there is a 25% dropout rate a total cohort of 1072 patients with will need to be recruited into the trial
Data collection	Data were collected from clinical notes and recorded in an electronic data capture platform (EDC). Patient satisfaction and answers to the EQ-5D-5L questionnaire were recorded on paper and via telephone (during the pandemic) and answers were entered onto the EDC. Following the scan, anonymised MR data were analysed off-site by specialised imaging analysts trained in abdominal anatomy and artefact detection who were blinded to all the clinical data.

Timing	Once they have had their scan, patients will be followed up for a period of 6-12 months.
Data exclusions	As autoimmune hepatitis is an exclusion criterion for MASLD/MASH diagnosis, one patient was excluded from analyses looking at biopsy avoidance.
Non-participation	There was high patient acceptance of the MRI scan resulting in only 1.7% (7/403) from the imaging arm (7/802 from the whole study) declining or being unable to have an MRI scan.
Randomization	Randomisation was done automatically using a random combination of inclusion criteria, and no significant differences in age, sex, weight, BMI biochemical markers or proportion with T2DM were observed between the two arms (Table 1).

Reporting for specific materials, systems and methods

We require information from authors about some types of materials, experimental systems and methods used in many studies. Here, indicate whether each material, system or method listed is relevant to your study. If you are not sure if a list item applies to your research, read the appropriate section before selecting a response.

Materials & experimental systems

n/a	Involved in the study
☒	☐ Antibodies
☒	☐ Eukaryotic cell lines
☒	☐ Palaeontology and archaeology
☒	☐ Animals and other organisms
☐	☒ Clinical data
☒	☐ Dual use research of concern

Methods

n/a	Involved in the study
☒	☐ ChIP-seq
☒	☐ Flow cytometry
☒	☐ MRI-based neuroimaging

Clinical data

Policy information about clinical studies

All manuscripts should comply with the ICMJE guidelines for publication of clinical research and a completed CONSORT checklist must be included with all submissions.

Clinical trial registration	NCT03289897
Study protocol	published protocol: Tonev D, Shumbayawonda E, Tetlow LA, Herdman L, French M, Rymell S, Thomaides-Brears H, Caseiro-Alves F, Castelo-Branco M, Ferreira C, Coenraad M, Lamb H, Beer M, Kelly M, Banerjee R, Dollinger M; RADIcAL1. The Effect of Multi-Parametric Magnetic Resonance Imaging in Standard of Care for Nonalcoholic Fatty Liver Disease: Protocol for a Randomized Control Trial. JMIR Res Protoc. 2020 Oct 26;9(10):e19189. doi: 10.2196/19189. PMID: 33104014; PMCID: PMC7652684.
Data collection	RADIcAL-1, a prospective, randomised controlled, multi-national trial, included patients recruited from 10 clinical centres in: Ulm (Germany), Leiden (Netherlands), Coimbra (Portugal) and 7 sites across the UK. European sites were set up in 2017 (with recruitment continuing up until 2020 in Ulm and Leiden) while UK sites joined the study at varying times between 2019 and 2020.
Outcomes	In this prospective, randomised controlled, multi-national European trial we investigated the impact on patient management including mpMRI in the MASLD clinical pathway would have in a real-world setting. We identified four key findings. Firstly, regarding the primary objective of this study, in a population with suspected MASLD, mpMRI is a cost-effective tool with an ICER of €4,929/QALY gained. Secondly, with regards to liver-related consultations, when using mpMRI as part of patient management, there are significantly less clinical consultations and follow-up appointments with healthcare specialists when compared to standard of care alone. Additionally, the proportion of patients with a final diagnosis by the end of the trial follow-up period was significantly higher in the imaging arm compared to SoC alone implying that mpMRI has a significant impact on rate of diagnosis. Third, mpMRI identified 50% of patients who did not require a biopsy for the diagnosis of MASH thereby showing clinical utility to support patient stratification for those with suspected MASLD. Lastly, there is an opportunity to optimise the clinical efficiency and long-term costs of MASLD clinical care as notable differences in real-world patient care highlight the need for streamlined management.
